# The A B C of Photo-Micrography

**Published:** 1904-01

**Authors:** 


THE A B C OF PHOTO-MICROGRAPHY. A Practical Hand-Book for Beginners. By W. H. Walmsley. 155 pages, 5x7, with 29 photo-micrographs by the author. Cloth, $1.25 net. Tennant & Ward, New York.

The lack of any American book dealing with this fascinating branch of photographic work, and the great need of an elementary introduction of photo-micrography, has led Mr. W. H. Walmsley to prepare this excellent manual. Mr. Walmsley is a recognized authority in the photo-micrographic world, and has had a more varied and longer experience in the field than most of his co-workers. He deals with his subject in a plain but comprehensive way, and the beginner who will study the A B C should find his difficulties vanish. The illustrations add largely to the practical value of the book and are, in themselves, most interesting.

